# Barriers to proper maternal referral system in selected health facilities in Eastern Ethiopia: a qualitative study

**DOI:** 10.1186/s12913-024-10825-3

**Published:** 2024-03-27

**Authors:** Betelhem Mengist, Agumasie Semahegn, Shegaye Yibabie, Bezabih Amsalu, Abera Kenay Tura

**Affiliations:** 1 Department of Midwifery, College of Medicine and Health Sciences, Dire Dawa University, Dire Dawa, Ethiopia; 2 School of Nursing and Midwifery, College of Health and Medical Sciences, Haramaya University, Harar, Ethiopia; 3 Center for Innovative Drug Development and Therapeutic Trials for Africa (CDT-Africa), College of Health Sciences, Addis Ababa University, Addis Ababa, Ethiopia; 4 Department of Population, Family and Reproductive Health, School of Public Health, University of Ghana, Accra, Ghana; 5 School of Medicine, College of Medicine and Health Sciences, Dire Dawa University, Dire Dawa, Ethiopia; 6 Department of Public Health, College of Medicine and Health Sciences, Dire Dawa University, Dire Dawa, Ethiopia; 7 Department of Obstetrics and Gynecology, University Medical Centre Groningen, University of Groningen, Groningen, the Netherlands

**Keywords:** Maternal referral practice, Barriers to proper referral, Eastern Ethiopia

## Abstract

**Background:**

Appropriate maternal referral system plays an essential role in curbing maternal mortality. Although the occurrence of obstetric complications is often unpredictable, addressing bottlenecks of the referral system is crucial to facilitate the women to have access to timely lifesaving interventions. Nonetheless, little is known about the barriers to maternal referral system in the eastern Ethiopia. Therefore, this study aimed to explore the barriers to maternal referral system at selected referral hospitals in eastern Ethiopia.

**Methods:**

Key informant interviews and in-depth interviews were conducted among purposively selected respondents who had a role in maternal referral processes. A total of 12 key informants that comprised of liaison officers, healthcare providers and three in-depth interviews with referred women were conducted. Semi-structured interview guide was developed and used to facilitate the interviews. All the interviews were tape recorded, complemented by note taking. Then audio recorded interviews were transcribed as per verbatim and imported to NVivo for coding and merging. The data were thematically synthesized.

**Results:**

The study identified a range of barriers that affect the maternal referral system in Eastern Ethiopia. The main barriers are grouped into three domains, such as: communication, transportation, and healthcare system. The most commonly reported barriers were lack of pre-referral communication and feedback, using informal communication, incomplete referral forms, poor ambulance service including misuse of ambulances, lack of skilled healthcare escort and lack of medical equipment at emergency, unnecessary self-referrals, poor referral skills and limited number of health professions.

**Conclusions:**

The maternal referral system is overwhelmingly affected by lack of skill, logistics (referral form), misuse of available ambulance, poor communication, and limited seeking of feedback. Regular consultative meeting with relevant stakeholders and uptake of feedback are crucial to improve referral communication, proper use of ambulance and building capacity of health workforce about referral are essential to improve maternal referral system.

**Supplementary Information:**

The online version contains supplementary material available at 10.1186/s12913-024-10825-3.

## Background

Maternal mortality is a major global public health agenda that requires complex interventions. Most of maternal deaths are due to unpredicted complication during pregnancy and childbirth, but preventable if diagnosed early [1]. Maternal mortality is unacceptably high, specifically in low- and middle-income countries (LMICs). About 295,000 women died during and following pregnancy and childbirth in 2017. The vast majority of these deaths (94%) occurred in LMICs but most could have been prevented [2], and sub-Saharan Africa alone accounted roughly two-thirds (196,000) of maternal deaths [3]. Majority of maternal mortalities are caused by direct obstetric events, such as hemorrhage, hypertension, obstructed labor, sepsis, eclampsia and complications of abortion [4]. Ending preventable maternal mortality can be achieved by making pregnancy and childbirth safer through helping women to have access to timely obstetric care [5]. Studies showed that Ethiopia was one of the countries that had more than 10,000 maternal deaths [6–8], with maternal mortality ratio ranging from 412 per 100,000 live births [9] to 543 per 100,000 live births [10].

Reducing maternal mortality ratio to less than 70 per 100,000 live births globally and no country has a maternal death more than 140 per 1000,000 live birth by 2030 is the key United Nations target under the Sustainable Development Goal (SDG 3) [11]. Availing women access to timely Emergency Obstetric Care (EmOC) remains as one of the major challenge in maternal health [12]. Proper maternal referral system plays an essential role in accessing better care to reduce maternal mortality [13]. Referral is a two-way process by which a health worker or health facility transfers the responsibility of care temporarily or permanently to another health professional or health facility in response to its inability to provide the necessary type of intervention suitable to the needs of the patient or due to shortage of resources [14].

Inappropriate maternal referral systems in LMICs are due to noncompliance with referral policy [15], poor documentation, lack of well-designed referral forms, healthcare providers burn out because of sluggish bureaucracies, no consultation or communication, poorly coordinated referrals between two facilities and working within inadequate resource at referral facilities [16–18], weak health information systems to capture referral data, and poor transport arrangements for emergency referrals [19].

Availing access to timely EmOC to women [1, 20], strengthening EmOC [21], and implementing effective referral system for obstetric complications [22, 23] have crucial contribution to prevent maternal mortality. The WHO has estimated that 88–98% of maternal morbidity and mortality can be averted through timely access to basic EmONC using appropriate referral system [2]. While the Ethiopian Ministry of Health has been investing in infrastructure, such as roads, ambulance services, and communication systems for referral [24] to improve the quality of maternal health service, the maternal referral system continued to suffer from barriers at different levels. The aim of this study was to explore barriers to proper maternal referrals among healthcare staff and referred mothers from selected health facilities, eastern, Ethiopia.

## Methods

### Study setting

The study was conducted at Hiwot Fana Comprehensive Specialized University Hospital (HFCSUH) and Dil Chora Referral Hospital (DCRH) found in Harar and Dire Dawa, respectively, Eastern Ethiopia. Harar is one of the oldest cities in Ethiopia and currently serving as a capital city of the Harari Regional State, located 525 km from east of Addis Ababa. Harar has an estimated population of 205,000, of which 54.8% were urban residents. Harari Region has 31 health posts, 8 health centers, 34 private clinics and 5 hospitals (4 governmental and 1 private hospitals) (Harari Regional Health Bureau, 2019). HFCSUH is a referral teaching hospital serving approximately 5.2 million people in Harar and surrounding communities from Oromia, Dire Dawa and Somali Regional states of Ethiopia (Human resource management, 2020). The obstetrics ward receives referred pregnant and laboring women from different health facilities. It has two sub-wards (labor and postnatal ward) including one triage room with a total of 63 beds. The obstetric ward was run by 15 consultants, 35 residents, 30 midwives, and 20 medical interns (human resource management of HFCSUH, 2021). Dil Chora Referral Hospital (DHRH) is located in Dire Dawa city administration 515 km from Addis Ababa in the eastern Ethiopia. In 2021 there were 2 public hospitals (DCRH and Sabian General Hospital), 4 private hospitals, 5 health centers, 15 higher clinics, 12 medium clinics, and 31 health posts serving approximately one million population from Dire Dawa and neighboring Oromia and Somali Regional States in Ethiopia. The obstetrics ward has two sub-wards (labor and postnatal wards) with a total of 39 beds. The obstetric ward was run by 4 consultants and 18 midwives (human resource management of DCRH, 2021).

### Study design and sampling

A qualitative study design was employed using phenomenological approach from July 01 to 31, 2021.

Obstetric care providers and liaison officers at the obstetrics ward of the hospitals were purposively selected for key informant interviews (KII) and in-depth interview (IDIs) of women who were referred from other facilities. The ward leaders were contacted to link with the selection of referred women who arrived and received care at referral hospitals. A total 9 key informants and 3 in-depth interviews were conducted to explore challenges for referral process.

### Data collection methods

A semi-structured interview guide was designed for qualitative study by adapting from existing relevant literatures [25–29], and contextualized to the local area. Questions listed in the interview guides were open ended and asked to liaison officers, referring health facility healthcare providers, receiving hospital healthcare providers, and referred patients on the referral of obstetrical emergency cases to the hospital. The interview guides primarily prepared in English and were translated into the local languages (Amharic and Affaan Oromo), and then back translated to English by an independent translator to ensure its consistency. Training was provided to the data collectors and supervisors about the data collection tools and collection procedure for two days. Supportive supervision was provided to data collectors by trained supervisors. All KIIs and IDIs were conducted by two trained interviewers. Healthcare providers and liaison officers were interviewed at their office or wards. Interviews with patients [women] were also conducted in their admitted wards. All interviews were tape audio recorded using voice recorder. All interviews with participants lasted between 45–60 min.

### Data management and analysis

Collected data were tape audio recorded and complemented with note taking by data collectors. All the data were transcribed using local languages as per the verbatim of study participants. Then transcribed data were translated into English language by experts. Then transcribed qualitative data were imported to NVivo for coding and management. All the contents of the transcripts were read, re-read and color coded, and themes were identified. Three major themes and 11 sub-themes were identified, and merging of codes was performed. Finally, compelling extracts of the data were used to back analysis in relation to the objectives of the study using thematic analysis method.

## Results

A total of 9 key informant interviews and 3 in-depth interviews were conducted. The majority of the health workers were midwives (Table 1). The study identified challenges encountered in the maternal referral system. Most of the identified challenges caused pregnant and laboring women’s delay in reaching health facilities and accessing healthcare. The challenges are presented thematically bellow.

**Table 1:** Basic socio-demographic characteristics of key informants/participants

**Participants**	**Profession**	**Position**	**Place of work**	**Age**	**Gender**	**Education**	**Experience**	**Unique ID**
Health worker	Midwife	Head of the ward	Referral hospital	35	F	MSc/maternity & Neo	16 years	M01
	Midwife	Head of the ward	Referral hospital	29	M	BSc/Midwifery	7 years	M02
	Nurse	Liaison officer	Referral hospital	26	M	BSc/Nursing	3 years	N01
	Nurse	Focal person	Referral hospital	40	M	BSc/Nursing	18 Years	N02
	Social worker	Liaison officer	Referral hospital	30	M	BSc/Social worker	6 years	N03
	Midwife	Staff	Referral hospital	32	M	MSc/maternity& Neo	7 years	M03
	Midwife	Staff	Referral hospital	36	F	BSc/midwifery	12 years	M04
	Midwife	Staff	Health center	32	F	BSc/midwifery	5 years	M05
	Midwife	Staff	Health center	35	F	BSc/midwifery	7 years	M06
**Participants**		**Occupation**	**Referring health facility**	**Age**	**Educational level**		**Marital status**	
Referred patients		Housewife	Rural health center	32	No formal education		Married	W01
		Housewife	Urban private hospital	26	Secondary school		Married	W02
		Housewife	Rural health center	28	No formal education		Married	W03

### Poor communication

Most of the study participants reported that the maternal referral challenged by poor communication and/or incomplete referral information. Mostly referred mother were sent using informal communication, such as phone call without sending a referral letter. Incomplete referral documents, poor pre-referral communication and poor feedback system were most frequently observed in referral system. Some referral papers did not contain the diagnosis, prior management(s) given, and details of the referring clinicians. One of the study participant remarked;

*“…Mostly referral letters did not contain information about the investigations, diagnosis, reason for referral, and pre-referral managements given. Therefore, we were forced to check from the patients if she had received any medication at the referring facility… I remembered asking a family member of an eclamptic woman whether she had a bilateral injection to assess whether a woman took loading dose of magnesium sulfate or not…” (M03)*

Another respondent stated;

*“…sometimes we have to call those health care providers to get the missed information of the referred women. However, some referrals did not have telephone numbers or other contact details. I think, this might be related to fear of blame, especially for inappropriate referral in the annual maternal health review meeting. So, they hide their contact details due to fear of professional critics or comment …’’ (M02)*

A lack of standard referral form/paper and/or training was the barrier to the maternal referral system. Another study participant has also stated:

*“…we are not using a standard referral slip. I have tried to write the pertinent information on in-house or locally prepared referral form when referring the patient since the referral slip prepared by our health facility does not have adequate space. In addition, many health care providers did not have training about referral system… that is why the referral form (documents) are incomplete most of the time …” (H05)*

### Lack of pre-referral communication

Most participants stated that there was no pre-referral communication between health facilities due to negligence or poor network connectivity.

*‘’…although referrals should be sent with a pre-referral communication, there is no pre-referral communication most of the time. I think, mostly it is due to poor internet or telecom network connectivity and sometimes negligence...” (N01)*

*“…most referrals from private clinics use informal referral letters and without pre-referral communication...” (M03)*

Most of the study participants agreed that poor communication between healthcare providers caused much sufferings to the referred patients.

*“… Some health facilities may try to make a pre-referral communication for one case; but they send two or three patients in one ambulance without any communication. It makes the hospital over busy with inappropriate referral…” (M01)*

*“…even if they are not sending the patient by ambulance, at least they should refer the women with appropriate pre-referral communication so that we will get well prepared to help the women if it is an emergency…” (N03)*

Majority of the study participants remarked that assigning hospital liaison officers without considering their clinical training background caused a communication barrier in the maternal referral system. One of the participants shared their experiences:

*“… I think there is also communication barrier at referral host health facility. Sometimes the liaison officers are not health professional ‘clinician’; so, they couldn’t understand what we are talking about. One day, I called a hospital to refer a woman who was suffering from PPH ‘postpartum hemorrhage’. Then, the person over the phone was saying ‘let me confirm if there is a bed. Otherwise, you don’t try to send the women’… imagine? …if he knew the case, he would never say this…” (M06)*

### Poor feedback system

Majority of health professionals reported that they would provide feedback to the referring facility through a liaison officer, nevertheless, most of the referral letters did not have enough space to write comprehensive feedback. Sometimes the feedback space may be filled end to end by the sender. Some feedback are given informally through referral escorts or ambulance drivers, and during supportive supervisions time. It also seems that some providers are tired off sending feedback to facilities. One of the studies participants reflected his concern as follow;

*“…what is the value of sending feedback if there would be no change? I am not sure whether the feedback properly reaches to the referring facilities healthcare provider or not. In addition, we are not sure if they are checking what we have written in the feedback section. On the other way, some of us did not even know what should be written in the feedback section. Most of the time, we are too busy so that we have no time to write very well-organized feedback… I think we are not doing well in that particular regard…” (M03)*.

Some social media platforms are so popular (e.g., Telegram) that may create a good opportunity for sending feedback easily.

*“…since some months back we created a telegram channel for maternal referral system. Whenever, we got unnecessary or inappropriate referrals, picture of the referral letter will be posted in that group chat telegram platform ‘naming and shaming’. The members in the telegram platform will write comments about it that can be taken as lesson by the referred facility or personnel …. I think there is some improvement because any healthcare provider has the responsibility to minimize inappropriate referral system.” (M01)*

### Lack of transportation

The major transportation challenges for the referral system are stated as poor ambulance services (lack of escort, lack of medical equipment at emergency)*,* inappropriate utilization of ambulance, transportation cost, and Poor infrastructure for women from rural health facilities.

### Poor ambulance services

Majority of the participants stated that even though patients are coming with ambulances, it is often not in the appropriate way including lack of accompanying healthcare providers and no life supportive equipment like oxygen administration, running intravenous transfusion and proper monitoring of patients on the way. A respondent stated; that.

*“… Ambulance alone has not been enough if there is no adequate medical equipment and paramedics for emergency. Almost all ambulances do not have oxygen… most women are coming without an accompany; if she bleeds on the way, no one can help her, as the driver or family members are not qualified for such help… therefore, the woman may die on the way in the ambulance before arrival. We are also observing some ‘accompanying healthcare providers sitting with the driver at the front seat…” (N03)*

Even though most health centers have ambulances, they are inappropriately used.

*“…at an emergency, when we ask for ambulance, we could not get. But we know that it is being used for other purposes… sometimes ambulances give service to local politicians ‘for non-medical service …” (M05).*

### Poor infrastructure

Some study participants reported that most of the mothers, who lived in the rural areas or far from the referring facilities, did not get ambulances or other forms of transportation due to road conditions. A healthcare professionals and referred woman shared their experiences:

*“…lack of transportation from home to health facility is the main barrier to maternal referral system of the women in the rural area. After arriving at a health facility, it is a little bit easy to refer the patient with ambulance. As a result, most of such women came with obstetrical complications too late…” (M06)*

*A woman who lost her baby due to late arrival sadly shared her experience as follows: “… I was bleeding; the health facility is far from my home and the road is difficult, I couldn’t get transportation to arrive the health facility in time… I used traditional transport means with the help of my family to reach to the nearby local health facility; Then, healthcare providers referred me to this hospital, but I have already lost my baby … I think I arrived to the health facility too late…” (W01)*

### Transportation cost

Some participants stated that women in the catchment area are sufferring from unnecessary payment to the ambulance drivers.

*“…it is known that ambulance services for pregnant or postpartum women are free. But I have seen those women who came from neighboring Regional Sates ‘Oromia and Somali’ paying the driver… I have seen when the referred women paid 2,000 Ethiopia Birr… I have tried to confirm this issue, and I am told that it is common…” (N01).*

### Self-referral

*“…mostly women came without a referral letter and they preferred to give birth at higher health facilities. But we are too busy and feel that normal labor should be managed at the primary health facilities… referral health facilities should not be busy by normal labors, because the tertiary facility should provide higher level care to women who really need. Being too much busy by normal labor may cause many obstetric complications…” (M04)*

*“…we can’t send back women with normal labor once they arrived here (referral hospital) because it is sensitive issue and also her right to health. We believe that she has the right to give birth wherever she likes…but self-referral may overburden for healthcare providers.” (N02)*

### Lack of necessary referral skills

Skill gap at lower health facilities was stated by many as follows:

*“...junior healthcare providers are deployed to work at rural health facility without having adequate skill to manage obstetric cases. Those are referring the women inappropriately …” (M02)*

*“…sometimes healthcare providers at lower health facilities didn’t know which case should be referred or not. They would sometimes refer a woman in labor with rupture of membrane as premature rupture of membrane (PROM)… most of such referrals gives birth in ambulance before arrival…” (M04)*

*“…since healthcare providers did not have adequate experience, most of them have just fear of complications. That is why they want to send the mother to the referral hospital unnecessarily…” (M03), and “… sometimes antepartum hemorrhage (APH) cases are being referred without IV-line…” (M04)*

### Shortage of health personnel

Shortage of skilled health personnel at referring facilities are also a reason for referring women without accompanying in the ambulance. It is also becoming a justification for inappropriate referrals.

*“…referring facilities do not have enough staff to escort the referred women. Some facilities may have only one midwife, especially at duty time… How a health facility with one midwife can give a comprehensive service to referred woman on the way ... It is difficult…” (M05)*

*“…especially at night duty time, most health workers are preferring to refer a woman immediately at arrival even before evaluating her…” (M03)*

### Delay to get care at receiving facilities

In addition to the challenges at the referring facilities, delays at receiving facilities were also reported as a challenge. A respondent remarked a recent tragic moment in their hospital:

*“… A woman arrived in our hospital with PPH. The midwife in charge was trying to consult a senior obstetrician over the phone. Although our office sent an ambulance to the senior obstetrician, the driver could not easily get the doctor [at ...home]… When the doctor reached the hospital, it was too late and the woman had already passed away. …” (N02)*

## Discussion

This study identified major barriers to the maternal referral system for emergency obstetric cases in Eastern Ethiopia. Incomplete referral information, poor pre-referral communication and poor feedback were described as the major challenges related maternal referral system.

Findings from this study mostly concentrated on the importance of communication between referring health facilities and receiving hospitals. The adequate provision of referral information is important to ensuring timely continuity of care at referred health facility. Patients referred to the unit were accompanied by referral slips but mostly them are incomplete. Again, emergency cases come without pre-referral communication and with poor referral letters. This finding is supported with studies conducted in Ghana and Ethiopia, which showed that poor communication is one the main challenges in referring maternal cases to the next health facility [27, 30, 31]. The initiation of mobile telephones was seen as vital to enhancing communication for referrals [32]. Several challenges were identified relating to communication between the referring facility and the receiving hospital. Such factors as poor network connectivity, negligence by health care providers and a lack of standard referral papers were supported to account for non-pre-referral communication on referrals. This finding is supported by narrative study conducted in developing country [33]. Health professionals recognized that the communication gap could be due to network connectivity challenges with some of the lower health facilities. However, the practice of referring complicated cases to higher facilities has also been seen as an attempt to escape the responsibility concerning pregnant woman from fear of being blamed by higher authorities in the likely event of adverse outcomes [27].

The major transportation challenges for the referral system are stated as poor ambulance services (lack of escort, lack of medical equipment at emergency*,* and unnecessary utilization of ambulance), financial barriers for transportation, and lack of means of transportation for district mothers. The FMOH has determined that it is imperative that all pregnant women have access to skilled attendance. Although, maternal services had been made free in the country, poor ambulance services for emergency obstetric cases were identified as a major challenge by all the respondents for the study. This however falls out on the prerequisites for a successful maternal referral systems as advanced by Murray and Pearson [19]. Similar findings were also noted in studies on emergency obstetric referrals in primary health facilities in Ethiopia and Ghana [27, 30, 31]. A qualitative study in rural Zambian and Tanzania also revealed that the unreliability of transport, distance and the high cost of referral are challenges for the maternal referral system [26, 34].

Though the availability of public and private transport may not be a problem in urban areas, the preference for using ambulance in transporting emergency cases is emphasized by safety and accessibility reasons. The use of ambulances helps to ensure continuity of care during the transit period by paramedics and also guard against delays in travelling through busy cities. This service is however troubled with several logistic challenges. Poor utilization of ambulance services according to most of the respondents were attributed to lack of escort, lack of medical equipment at emergency, and unnecessary utilization of ambulance for other purposes. Poor ambulance service as reported by some healthcare providers should be addressed as a similar finding has shown to affect emergency obstetric care in a teaching hospital in Nigeria and Ghana [27, 35]. The study showed that the major obstacles to maternal referral were unnecessary referrals, self-referrals, gap in referral skills, and shortage of staff. Similar findings were also noted in Iran and Kenya [28, 36].

### Study strength and limitation

This study provides information on the referral of obstetric emergencies to the referral hospitals in eastern Ethiopia. It brings to an understanding of the barriers to maternal referral system using multiple stakeholder perspectives. The finding may not be generalized to the overall community and health facilities due to the nature of sampling procedure of qualitative study sampling.

## Conclusions

Maternal referral system in eastern Ethiopia has many inappropriate practices that impact the lives of pregnant and laboring women. The referral system was stranded into poor communication, logistics scarcity, transportation and healthcare personnel capacity. Lack of pre-referral communication and feedback, using informal communication, incomplete referral documents, lack of transportation, misuse of ambulances, lack of essential medical equipment at emergency, unnecessary self-referrals, poor referral skills, limited number of health professionals, and lack of skilled healthcare escort were the major barriers. We suggest the interventions are crucial to improve misuse of ambulance, establish both formal and informal communication network, building capacity of healthcare providers on referral management and deploy appropriate liaison officers. 

### Supplementary information

**Supplementary Material 1.**

## Data Availability

The data sets used for this study are available from the corresponding author (Bezabih Amsalu, mamtnur100@gmail.com) on reasonable request.

## Abbreviations

DCRH: Dil Chora Referral Hospital
EmOC: Emergency Obstetric Care
HCP: Health Care Provider
HFCSUH: Hiwot Fana Comprehensive Specialized University Hospital
IDI: In-depth Interview
IHRERC: Institutional Health Research Ethics Review Committee
KII: Key Informant Interviews
LMICs: Low and Middle-Income Countries
MOH: Ministry of Health
NGO: Non-Governmental Organizations
PHC: Primary Health Care
SDGs: Sustainable Development Goals
WHO: World Health Organization
