# Supplementary material for: Barriers to proper maternal referral system in selected health facilities in Eastern Ethiopia: a qualitative study
Source: BMC Health Serv Res. 2024 Mar 27;24:376. doi: 10.1186/s12913-024-10825-3 (PMC10967082; doi:10.1186/s12913-024-10825-3)
Supplement: Supplementary file 1 — Supplementary Material 1. [file 12913_2024_10825_MOESM1_ESM.docx]

## Participants’ Information Sheet and Voluntary Consent Form for participants of key informants

My name is ____________________________. I am working as data collector for the study being collected by Betelhem Mengist who is the principal investigation of this research and pursing her postgraduate education at Haramaya University, College of Health and medical Sciences. I kindly request you to help me by giving information about the study and being selected as the study participant.

**Title: Barriers to proper maternal referral system of selected health facilities in Eastern Ethiopia: a qualitative study**

**Purpose of the study**: The findings of this study will be aimed for Dire Dawa and Harar town Regional Health Bureau and health facilities to plan intervention programs to reduce inappropriate maternal referral system.

**Procedure and duration**: I will be interviewing you using questionnaire to provide me with pertinent data that is helpful for the study. There are 10 questions to be answered where I will fill the questionnaire by interviewing you. The interview will take about 45 -60 minutes, so I kindly request you to spare me this time for the interview.

**Risk and benefits**: The risk of being participated in this study is very minimal, but only taking few minutes from your time. There would not be any direct payment for participating in this study but the findings from this research may reveal important information for communities and health facilities around Dire Dawa and Harar cities where the two referral hospitals located and inform, Regional Health Bureaus and health facilities future planning.

**Confidentiality**: The data you will provide us will be kept confidential. There will be no information that will identify you in particular. The findings of the study will not reflect anything particular of individual person. No reference will be made in oral or written reports that could link participants to the research.

**Rights**: Participation in this study is fully voluntary basis. You have the right to declare to participate or not in this study. If you decide to participate, you have the right to withdraw from the study at any time during the interview and this will not label you for any loss of benefit which you otherwise are entitled. You do not have to answer any question that you do not want to answer.

**Contact address**: If there, are any questions or enquires any time about the study or the procedures, please contact me:

Betelhem Mengist: Mobile number (+251946459915)

Email Address: betelhemg8@gimal.com

Institutional Health Research Ethics Review Committee: Phone Number (+251)-025-466-07-08, P.O.Box 235, Harar

**Declaration of informed voluntary consent:** I have read/was read to me the participant information sheet. I have clearly understood the purpose of the research, the procedures, the risks and benefits, issues to confidentiality, the rights of participating and contact address for any queries. I have been given the opportunity to ask questions for things that may have been unclear. I was informed that I have the right to stop the study at any time or not to answer any question that I do not want. Therefore, I declare my voluntary consent to allow this study to be conducted with my initials (signature).

Name and signature of the participant: ______________________

Name and Signature of data collector: ______________________

N.B: **This is to be signed face to face in the presence of data collectors**

**Please provide a copy of this signed consent to the participant.**

## Qualitative Interview Guide for key informants

Data collector’s Name: ________________________Signature: __________ Date ___________

Supervisor’s Name: ________________________ Signature: ________________________

Code No ______________ Hospital/health center Name: ___________________

**Introduction**:

Thank you very much for agreeing to do this interview with me today. We are interested in learning about your opinions about the barriers to proper maternal referral system of health facilities referral system for pregnant and laboring women. This interview will mainly target key informants from **health facility heads, or liaison** **officers, and health care providers.**

**Interview guide for heads of the health facility and liaison**

1. Age ………………. Sex …………
2. Educational level …………. (Diploma, Degree, MSc, Medical specialty, other specify…).
3. Profession ……………….. (MD, Health officer, Midwife, Nurses, IESO, Other (specify……)
4. Work Experience ___________ (in Year)
5. Would you explain your role in this facility? ………………………

Probe: for how long do you work in this position…. so what ….., activities related with referral

1. Does the health facility have focal person for referral? ........................................................... Probe: how it facilitates the referral system? (Timely communication with HCP, Communication with receiver hospital and arrange transportation)
2. Would you explain the mode of transportation for referred woman? .............................. (by what, how, when, by their own payment, public transportation, referral guidelines )

Probe: Tell me more about the ambulance service

1. Tell me your experience how you communicate with the receiver facility? ………

probe: What are the communication options with the receiver facilities(Referral paper, Telephone, network, Telegram, Email, Posta and)? ………………

1. Would you explain your experience related to referral form and system? ..........................

Probe: How does health facility use it?, training, Avail referral guidelines, human resource ……..

1. Would you tell me about feedback? ……………………………………………

Probe: when you send the feedback? How you send the feedback? How you check if the feedback is arriving, response…..)

**Interview Guide for referred mother**

Data collector’s Name: ________________________Signature: __________ Date ___________

Supervisor’s Name: ________________________ Signature: ________________________

Code No ______________ Name of referred hospital/health center ____________

**Introduction**:

Thank you very much for agreeing to do this interview with me today. We are interested in learning about your opinions about the barriers to proper maternal referral system of health facilities referral system for pregnant and laboring women. This interview will mainly target mother referred from other health facility.

**Age:- ……… occupation …………………. Education …………. Marital status ………..**

1. Would you tell me from where you came?
2. How do you come here? (Transportation)

**Probe**: how, by what, when (transportation, payment…)

Would you tell me about your perception of the ambulance service?

1. Have you faced any challenge during the referral process? What were those challenges?
2. were you well aware about why you are referred?

**Probe:** How can you communicate with a care provider or health facility if you are concerned about your health during pregnancy?

1. How do you see the referral system?
2. Would you tell me your experience related with referral system?

**Probe**: Challenges (at referring facility, on transportation receiver hospital …

የአማርኛ ቅጂ የተሣታፊ መረጃ ወረቀት እና የፍቃደኝነት ፈቃድ ቅጽ ለቁልፍ መረጃ ሰጭዎች ተሳታፊዎች

ስሜ……………………እባላለሁ፡፡አሁን እየሰራሁኝ ያለሁት በዚህ ማህበረሰብ ለሚደረገው ጥናት መረጃ ሰብሳቢ ሆኜ ለወ/ሮ ቤተልሔም መንግስት በሐረማያ ዩኒቨርሲቲ በእናቶችና ህጻናት በማስተርስ ደረጃ ለመመረቂያ የሚሆን ጥናት ለማካሔድ ነው፡፡ ስለዚህ እንዴት ተሳታፊ መሆን እንደቻሉና ስለጥናቱ በተመለከተ ማብራሪያ እንድሰጥዎት የተወሰነ ጊዜ እንዲሰጡኝ በአክብሮት እጠይቃለሁ፡፡

**የጥናቱርዕስ**፡ በምስራቅ ኢትዮጵያ ለተመረጡት የጤና ተቋማት ትክክለኛ የእናቶች ሪፈራል ስርዓት እንቅፋት፡

**የጥናቱ ዓላማ፡** የዚህ ጥናት ግኝት በምስራቅ ኢትዮጵያ ሪፈራል ሆስፒታሎች የነብሰጡር እናቶችን ቅብብሎሽ ለማሻሻል ለሚደረጉ ጥረቶች ከፍተኛ ጠቀሜታ ይኖረዋል፡፡ ከዚህ በተጨማሪም ለዋና አጥኚው የማስተርስ ትምህርቱን ለማጠናቀቅና የመመረቂያ ጽሁፍ ለማዘጋጀት ይጠቅመዋል፡፡

**የጥናቱ ሂደት እና ጊዜ፡** ለጥናቱ የሚያገለግሉና መረጃ ሊሰጡ የሚችሉ ጥያቄዎች ተዘጋጅተዋል፡፡ እነዚህ ጥያቄዎች ጠቅላላ 10 ሲሆኑ በቃለ-ምልልስ ጥያቄዎቹን ለመመለስ 45-60 ደቂቃ ይፈጃል፡፡ ስለዚህ አሁንም በድጋሚ ጊዜዎትን እንዲሰጡኝ በአክብሮት እጠይቃለሁ፡፡

**ጉዳትና ጥቅም፡** በዚህ ጥናት መሳተፍዎ ትንሽ ጊዜዎትን ከመውሰድ በስተቀር የሚደርስብዎት ጉዳት በጣም አነስተኛ ነው፡፡ በዚህ ጥናት በመሳተፍዎ የሚያገኙት ቀጥተኛ ጥቅም የለም፡፡ ነገር ግን ከጥናቱ የተገኙት ጠቃሚ መረጃዎች ስለ ጤና እና ጤናን በተመለከተ ለሚያቅዱ የሚመለከታቸው ባለድርሻ አካላት ይጠቅማቸዋል፡፡

**ምስጢር አጠባበቅ፡** የሚሰጡን መረጃ ሁሉ ምስጢራዊነቱ የተጠበቀ ነው። ለዚሁም እርስዎን የሚገልጽ ምንም ነገር የለም፡፡ የጥናቱ ውጤት በግለሰብ ሳይሆን ለሁሉም ህዝብ ነው፡፡ ጥያቄው መለያ ምልክት አለው፤ ስም የሚገጽ ነገር የለውም፡፡ እናም ስለተሳታፊዎች የሚገልጽ የቃልም ይሁን የጥሁፍ በጥናቱ ውስጥ የለም።

**የተሳታፊውመብት፡** በዚህ ጥናት ለመሳተፍ ሙሉ ፈቃደኝነት ያስፈልጋል፡፡ በዚህ ጥናት የመሳተፍ ወይም ያለመሳተፍ ሙሉ መብትም አለዎት፡፡ ላለመሳተፍ ከፈለጉ ደግሞ በማንኛውም ጊዜ በመሀል ራስዎን ከጥናቱ ማግለል(ማቋረጥ) ይችላሉ፡፡ ካቋረጥኩኝ ጥቅም ይጎልብኛል ብለው አያስቡ፡፡ መመለስ የማይፈልጉትን ማንኛውም ጥያቄ አለመመለስ መብትዎ ነው፡፡

**አድራሻ፡** ስለጥናቱ አካሄድ ወይም ስለጥናቱ መጠይቅ ወይም ደግሞ ጥናቱን በተመለከተ ማንኛውም ጥያቄ ካለዎት የሚከተሉትን አድራሻ ይጠቀሙ፡፡

ቤተልሔም መንግስት: ስልክ-+251946459915 ኢሜይል- betelhemg8@gmail.com

ተቋማዊ የጤና ምርምር ስነ-ምግባር ግምገማ ኮሚቴ፡ ስልክ-(+251)-025-466-07-08፣ፖ.ሳ.ቁ-235 ሀረር

**በፈቃደኝነት ላይ የተመሰረተ የስምምነት ማረጋገጫ፡** የተሳታፊውን መረጃ ፎርም አንብቤዋለሁ ወይም ተነቦልኛል፡፡ የጥናቱ ዓላማ፤ ያለውን ጉዳትና ጥቅም፤ ምስጢር አጠባበቅ የመሳተፍ እና ያለመሳተፍ መብት እንዲሁም ችግር ካለ ከማን ጋር መገናኛኘት እንዳለብኝ ሁሉ ተገልጾልኛል፤ ጥያቄ ካለኝ ደግሞ እንድጠይቅ እድል ተሰጥቶኝ በመሀል ደግሞ ጥናቱን ለማቆም ከፈለኩኝ በማንኛውም ጊዜ ከጥናቱ/ከተሳታፊነት/ መውጣት እንደምችል በመጨረሻም መመለስ የማልፈልገውን ጥያቄ አለመመለስ መብቱ እንዳለኝ ከተረዳሁኝ በኋላ በሙሉ ፈቃደኝነት በዚህ ጥናት ለመሳተፍ የወሰንኩኝ መሆኔን ከዚህ በታች በተቀመጠው ፊርማዬ አረጋግጣለሁ፡፡

የተሳታፊ ስም …………………………………ፊርማ ………………ቀን……………………

የመረጃ ሰብሳቢ ስም …………………… ፊርማ ………………. ቀን………………………………

**ለቁልፍ መረጃ ሰጭዎች የቀረበ የቃለ መጠይቅ መመሪያ**

የውሂብ ሰብሳቢው ስም፡ ________________________ ፊርማ፡ __________ ቀን ___________

የተቆጣጣሪ ስም፡ ________________________ ፊርማ፡ ________________________

ኮድ ቁጥር ______________ ሆስፒታል/የጤና ጣቢያ ስም፡_________________

**መግቢያ፡-**

ዛሬ ከእኔ ጋር ይህንን ቃለ ምልልስ ለማድረግ ስለተስማማችሁኝ በጣም አመሰግናለሁ። ለነፍሰ ጡር እና ምጥ ላይ ላሉ ሴቶች ትክክለኛ የእናቶች ሪፈራል ስርዓት የጤና ተቋማት ሪፈራል ስርዓት እንቅፋቶችን በተመለከተ የእርስዎን አስተያየት ለማወቅ ፍላጎት አለን። ይህ ቃለ መጠይቅ በዋናነት ከጤና ተቋም ኃላፊዎች፣ ወይም የግንኙነት መኮንኖች፣ እና የጤና እንክብካቤ አቅራቢዎች ቁልፍ መረጃ ሰጪዎችን ያነጣጠረ ነው።

**ለጤና ተቋሙ ኃላፊዎች እና ግንኙነት ኃላፊዎች የቃለ መጠይቅ መመሪያ**

1. ዕድሜ …………………. ጾታ …………

2. የትምህርት ደረጃ …………………. (ዲፕሎማ፣ ዲግሪ፣ ኤምኤስሲ፣ የህክምና ስፔሻሊቲ፣ ሌላ ይግለጹ…)።

3. ሙያ …………………. (MD፣ የጤና መኮንን፣ አዋላጅ፣ ነርሶች፣ IESO፣ ሌላ (ይግለጹ……)

4. የስራ ልምድ ___________ (በዓመት)

5. በዚህ ተቋም ውስጥ ያለዎትን ሚና ያብራራሉ? …………………………………………………. **ምርመራ፡** በዚህ ቦታ ለምን ያህል ጊዜ ሰርተሃለ?…. እና ምን….፣ ከሪፈራል ጋር የተያያዙ እንቅስቃሴዎች

6. የጤና ተቋሙ ለሪፈራል ፎካል ሰው አለው ወይ? ................................................. .........

**መርማሪ፡** የሪፈራል ስርዓቱን እንዴት ያመቻቻል? (ከኤች.ሲ.ፒ. ጋር ወቅታዊ ግንኙነት፣ ከተቀባይ ሆስፒታል ጋር መገናኘት እና መጓጓዣን ማዘጋጀት)

7. በሪፈራል ለሚላኩ ሴቶች የመጓጓዣ ዘዴን ብታብራሩልን? ................................... (በምን ፣ እንዴት ፣ መቼ ፣ በራሳቸው ክፍያ ፣ የህዝብ ማመላለሻ ፣ ሪፈራል መመሪያዎች)

**ምርመራ፡** ስለ አምቡላንስ አገልግሎት የበለጠ ንገረኝ።

8. ከተቀባይ የጤና ተቋሙ ጋር እንዴት እንደሚግባቡ ልምድዎን ይንገሩኝ? ………

**ምርመራ፡** ከተቀባዩ የጤና ተቋሙ ጋር ያለው የግንኙነት አማራጮች ምንድናቸው? ………… (ሪፈራል ወረቀት፣ ስልክ፣ ኔትወርክ፣ ቴሌግራም፣ ኢሜል፣ ፖስታ እና) ………

9. ከሪፈራል ቅፅ እና ስርዓት ጋር የተያያዘ ልምድዎን ያብራራሉ? .................................

**ምርመራ፡-** የጤና ተቋም እንዴት ነው የሚጠቀመው?፣ (ስልጠና፣ የሪፈራል መመሪያዎች፣ የሰው ሃይል)………………..

10. ስለ ግብረ መልስ ንገረኝ? …………………………………………………

**ምርመራ፡** ግብረ መልስ ስትልክ? ግብረ መልስ እንዴት እንደሚልኩ? ግብረ መልስ እየደረሰ መሆኑን እንዴት እንደሚፈትሹ ምላሽ ይስጡ…..)

**በሪፈራል ለመጣች እናት የቃለ መጠይቅ መመሪያ**

የውሂብ ሰብሳቢው ስም፡ ________________________ ፊርማ፡ __________ ቀን ___________

የተቆጣጣሪ ስም፡ ________________________ ፊርማ፡ ________________________

ኮድ ቁጥር ______________ የተጠቀሰው ሆስፒታል/ጤና ጣቢያ ስም ____________

**መግቢያ፡-**

ዛሬ ከእኔ ጋር ይህንን ቃለ ምልልስ ለማድረግ ስለተስማማችሁኝ በጣም አመሰግናለሁ። ለነፍሰ ጡር እና ምጥ ላይ ላሉ ሴቶች ትክክለኛ የእናቶች ሪፈራል ስርዓት የጤና ተቋማት ሪፈራል ስርዓት እንቅፋቶችን በተመለከተ የእርስዎን አስተያየት ለማወቅ ፍላጎት አለን። ይህ ቃለ መጠይቅ በዋነኝነት የሚያተኩርዉ ከሌላ የጤና ተቋም በሪፈራለ የተላከችን እናት ነው።

ዕድሜ፡- ሥራ …………………………. ትምህርት …………. የጋብቻ ሁኔታ ………..

1. ከየት እንደመጣሽ ንገረኝ?

2. እንዴት ወደዚህ የጤናተቋም መጡ? (መጓጓዣ)

**ምርመራ፡** እንዴት፣ በምን፣ መቼ (መጓጓዣ፣ ክፍያ…)

ስለ አምቡላንስ አገልግሎት ያለዎትን አመለካከት ይንገሩኝ?

3. በሪፈራል ሂደቱ ወቅት ተግዳሮቶች አጋጥመውዎታል? እነዚያ ተግዳሮቶች ምን ነበሩ?

4. ለምን ወደዚህ የጤና ተቋም በሪፈር እንደተላኩ በደንብ ያውቁ ነበር?

**ምርመራ፡-** በእርግዝና ወቅት ስለ ጤናዎ የሚጨነቁ ከሆነ ከእንክብካቤ ሰጪ ወይም ከጤና ተቋም ጋር እንዴት መገናኘት ይችላሉ?

5. የሪፈራል ስርዓቱን እንዴት ያዩታል?

6. ከሪፈራል ሲስተም ጋር የተያያዘ ልምድሽን ንገሪኝ?

**ምርመራ፡** ተግዳሮቶች (በማጣቀሻ ተቋም፣ በትራንስፖርት ተቀባይ ሆስፒታል ላይ…)

**Waraqaa Odeeffannoo Hirmaattotaa fi Unka fedhiin Hayyamamaa ta’uu hirmaattoota , odeeffattoota ijoo ta’aniif**

Maqaan koo ____________________________. Jedhamaa , ga’een koo Qorannoo sassaabamaa jiruuf qoratuunolaanaa qorannoo kanaa kan taate fi barnoota ishee digrii lammaffaa Yunivarsiitii Haramaya, Kolleejjii Fayyaa fi Saayinsii yaalaa keessatti hordofaa kaan jirtu B/tu Betelhem Mengist tiif raga sassaabaa ta’een hojjechaan jira. Waa’ee qorannichaa odeeffannoo naf kennuu fi hirmaataa qorannichaa ta’uun akka na gargaartan kabajaan isin gaafadha.

**Mata duree: Danqaawwan sirna rifaralaa haadholii sirrii ta’e dhaabbilee fayyaa Baha Itoophiyaa kessa kaan filatamaanii kesati geegeefamee : Akkamta Ibsa.**

**Kaayyoo qorannichaa:** Argannoon qorannoo kanaa Biiroon Eegumsa Fayyaa Naannoo Dire Dawaa fi Magaalaa Harar fi dhaabbileen fayyaa sirna rifaralaa haadholii hin malle hir’isuuf sagantaa sirreeffama karoorfachuufi kan kaayyeffate ta’a

**Adeemsaa fi yeroo:** Odeeffannoo barbaachisaa qorannichaaf gargaaran naaf kennuudhaaf gaaffilee fayyadameen isin gaafadha. Gaaffileen 10n deebii argachuu qaban, gaaffii fi deebii isiniif godheen guuta. Gaaffii fi deebiin gara daqiiqaa 45–60 waan fudhatuuf yeroo kana gaaffii fi deebiidhaaf akka naaf heyyamitan kabajaan siin gaafadha

**Balaa fi faayidaa:** Balaan qorannoo kana irratti hirmaachuu baayyee xiqqaadha, garuu yeroo keessan keessaa daqiiqaa muraasa qofa fudhata. Silaa qorannoo kana irratti hirmaachuuf kaffaltiin kallattiin hin jiraatu ture, garuu argannoowwan qorannoo kana irraa argaman hawaasaa fi dhaabbilee fayyaa naannoo magaalota Dire Dawaa fi Harar, bakka hospitaalonni rifaralaa lamaan jiraniif odeeffannoo barbaachisaa ta’e mul’isuu, akkasumas, biiroolee fayyaa naannoo fi dhaabbilee fayyaa waa’ee karoora gara fuula duraa.

**Mirga**: Qo’annoo kana irratti hirmaachuun guutummaatti fedhii ofiitiin kan hundaa’edha. Qo’annoo kana irratti hirmaachuu fi dhiisuu kee labsuuf mirga qabda. Yoo hirmaachuuf murteessite, yeroo af-gaaffii yeroo barbaaddetti qo’annoo keessaa ba’uuf mirga qabda, kunis faayidaa kasaaraa karaa biraatiin argachuu qabdu kamiyyuu addatii si hin dhabissisu. Gaaffii deebii kennuu hin barbaanne kamiyyuu deebisuun mirga keetii.

**Teessoo quunnamtii**: gaaffiin kamiyyuu waa’ee qorannichaa ykn hojimaataa saan walqabte Yoo jiraate, yeroo kamiyuu, maaloo na qunnamaa:

Betelhem Mengist: Lakkoofsa bilbila harkaa (+251946459915)

Imeelii: betelhemg8@gimal.com

Koree Gamaaggama Naamusa Qorannoo Dhaabbilee Fayyaa Morra Harar: Lakkoofsa Bilbilaa (+251)-025-466-07-08, P.O.Box 235, Harar

Labsii hayyamamaa fedhiin hirrimachuu ilaalichisee beekumsa qabchuu: Waraqaa odeeffannoo hirmaataa dubbiseera ykn naaf dubbifameera. Kaayyoo qorannichaa, hojimaata, balaa fi faayidaa, dhimmoota iccitii qaban, mirga hirmaachuu, fi teessoo quunnamtii gaaffii kamiyyuu sirriitti hubadheera. Wantoota ifa ta’uu dhiisuu danda’an irratti gaaffii akkan gaafadhu naaf hayyamameera. Yeroo barbaadetti qo’annoo dhaabuu ykn gaaffii ani hin barbaanne kamiyyuu deebisuu dhisuuf mirga akkan qabu naaf himameera. Kanaaf, qorannoon kun qubee jalqabaa (mallattoo) kootiin akka gaggeeffamu hayyamuuf fedhii kootiin hayyama koo nan ibsa.

Maqaa fi mallattoo hirmaataa: ______________________ .

Maqaa fi Mallattoo walitti qabaa odeeffannoo: ________________ .

N.B: Kunis bakka namoonni odeeffannoo walitti qaban jiranitti fuula fuulatti mallattaa’uu qaba

Maaloo hayyama mallattaa’e kanaa waraabbii hirmaataaf kenni.

**Qajeelfama Af-gaaffii Akkamta Odeeffattoota Ijoo**

Maqaa walitti qabaa odeeffannoo: __________Mallattoo: __________ Guyyaa

Maqaa Suppervaayizaraa: __________________ Mallattoo: ________________________ .

Lakk koodii ______________ Hospitaala/bufataa fayyaa Maqaa: ___________________ .

**Seensa**:

Har’a Af-gaaffii fi deebii kana na waliin gochuuf hayyamamaa ta’uu keessaniif baay'ee galatoomaa. Dhaabbilee fayyaa dubartoota ulfaa fi da’umsaa keessatti sirna rifaralaa haadholii sirrii ta’e akka hin jiraanne gufuulee jiran ilaalchisee yaada keessan baruuf fedhii qabna. Af-gaaffiin kun irra caalaa hoggantoota dhaabbilee fayyaa, ofisaroota qunnamtii, fi kennitoota eegumsa fayyaa dabalatee odeeffattoota ijoo irratti kan xiyyeeffatu ta’a.

**Qajeelfama af-gaaffii hoggantoota dhaabbata fayyaa fi qunnamtii**

1. Umurii ................... Saalaa ..........

2. Sadarkaa barnootaa..... (Dippiloomaa, Digirii, MSc, Medical specialty, kanneen biroo (ibsu...)

3. Ogummaa…….. (MD, Health officer, Midwife, Nurses, IESO, kanneen biroo (ibsu ……)

4. Muuxannoo Hojii ___________ (Waggaan) .

5. Gahee ati dhaabbata kana keessatti qabdu ni ibsita? .................................................................. **Gafii Itiifuufinsaa:** yeroo hammamiif bakka kana kessatti hojjettee.... kanaaf hojiiwwan rifaralaa wajjin kaan walqabatan

6. Dhaabbanni fayyaa kessan rifaralaan walqabetee nama bakka bua’a ta’e qabaa? …………

**Gafii Itiifuufinsaa**: sirna rifaralaan walqabatee akkamitti haala safissafi se’ataa ta’ee mijeessa? (HCP waliin yeroodhan walqunnamtii, walqunnamtii Hospitaala fudhataatuu waliin, fi geejjibaa qopheessuun)

7. Haala geejjibaa dubartii rifaralii ta’sifameef ni ibsita? .............................. (maal, akkamitti, yoom, kaffaltii isaaniitiin moo, geejjiba uummataan moo, qajeelfama rifaralaa) .

**Gafii Itiifufinsaa**: Waa'ee tajaajila ambulaansii caalaatti nafii ibisuu ni dandeessa?

8. Akkaataa dhaabbata referrala fudhataan wajjin itti wal qunnamtan muuxannoo keessan nafii ni ibisuu ni dandeessa?

**Gafii Itiifufinsaa**: Filannoon qunnamtii meeshaalee fudhataa (waraaqa referralaa, bilibilaa, walitii dhufeynaa, Telegram, imelii, Posta fi) waliin maal fa’a? ..................

9. Muuxannoo unka rifaralaa fi sirnaa waliin walqabatee qabdu ibisuu ni dandeessa? …………

**Gafii Itiifufinsaa**: Dhaabbanni fayyaa akkamitti itti fayyadama?, leenjii, Qajeelfama rifaralaa qophessudhan, humna namaan ........

10. Waa'ee yaada deebii kennuu natti himuu ni dandeessa? ......................................................

**Gafii Itiifufinsaa**: yaada Duub-deebii yerroo ergitu? Yaada Duub-deebii akkamitti ergitu?, yaada Duub-deebii ga’uu saa akkamin mirkaneefatuu , deebii.....)

**Qajeelfama Af-gaaffii haadholii rifaralaa ta’aniif**

Maqaa walitti qabaa odeeffannoo: ____________Mallattoo: __________ Guyyaa ___________ .

Maqaa Suppervaayizaraa:_______________________ Mallattoo: ________________________

Lakk koodii ________ Maqaa hospitaala/bufataa fayyaa referralii tasifamedhaaf___________

**Seensa**:

Har'a Af-gaaffii fi deebii kana na waliin gochuuf hayyamamaa ta’uu keessaniif baay'ee galatoomaa. Sirna rifaralaa haadholii sirrii ta’e sirna rifaralaa dhaabbilee fayyaa dubartoota ulfaa fi da’umsaa fi dhufanii fi (ccinisuuraa jiran) ilaalchisee yaada keessan baruuf fedhii qabna. Af-gaaffiin kun irra caalaa haadholii dhaabbata fayyaa biraa irraa rifaralaa ta’an irratti kan xiyyeeffatu ta’a.

Umurii:- ........... hojii ...................... Barumsa …………. Haala gaa'elaa ...........

1. Eessaa akka dhufte natti himtaa?

2. Akkamitti as dhufta? (Geejjibaan?)

**Gafii Itiifufinsaa**: akkamitti, maaliin, yoom (geejjibaa, kaffaltiin...)

Ilaalcha tajaajila ambulaansiin walqabatee yaddaa qabdan natti himuu nii daaniidessuu?

3. Adeemsa rifaralaa keessatti rakkoon isin mudataan jiruu?Rakkoowwan sun maal faa turan?

4. Maaliif akka referriin siif tasiifamee sirriitti beektaa turtee?

**Gafii Itiifufinsaa**: Yeroo ulfaa waa’ee fayyaa keetii yoo yaaddoon siraa ga’ee akkamitti ogeessa kunuunsaa fayyaa ykn dhaabbata fayyaa wajjin wal qunnamuu ni dandeessa?

5. Akkataa Sirna rifaralaa kenamaa jiruu akkamitti ilaaltu?

6. Muuxannoo keessan sirna rifaralaa wajjin walqabatuu qabdaan natti himuu ni dandeessu?

**Gafii Itiifufinsaa**: Danqawwan jiraan (bakka dhabilee rifaralaa, geejjibaa hospitaala fudhatuu, irratti ...)
